# Intramural Hematoma of the Duodenum in a Four-Year-Old Child Following a Blunt Bicycle Handlebar Injury: A Case Report

**DOI:** 10.7759/cureus.85113

**Published:** 2025-05-30

**Authors:** Olga Gryszkiewicz, Marek Wolski

**Affiliations:** 1 Department of Paediatric Surgery, Medical University of Warsaw, Warsaw, POL

**Keywords:** blunt injury, hematoma of duodenum, non-operative, pediatric, trauma

## Abstract

Duodenal hematomas are an uncommon occurrence in the pediatric population, most frequently resulting from blunt abdominal trauma. The preferred treatment modality is a non-operative management, which typically includes bowel rest, nasogastric tube, parenteral nutrition, serial laboratory evaluations, and follow-up imaging. Surgical intervention is rare and generally reserved for cases with complications or failure of conservative therapy.

Clinical presentation often includes abdominal pain, nausea, and vomiting. Due to the retroperitoneal location of the duodenum, physical examination findings are typically non-specific. Furthermore, imaging studies may yield false-negative results, contributing to delayed diagnosis and increased risk of complications.

In this case report, we present a four-year-old patient diagnosed with a large intramural duodenal hematoma and successfully managed with a conservative, non-operative approach.

## Introduction

Duodenal hematoma is an uncommon injury in the pediatric population, representing approximately 3%-5% of all abdominal traumas [1]. These injuries are frequently associated with concomitant damage to other abdominal or thoracic organs, including major blood vessels [2].

In contrast to adults where more than 70% of duodenal injuries result from penetrating trauma, the majority of duodenal injuries in children result from blunt injuries [3]. Pediatric anatomical features, such as a more horizontally oriented costal margin and relatively low intra-abdominal fat content, increase susceptibility to duodenal injury from blunt trauma [4].

Although such injuries may appear minor initially, delayed diagnosis is associated with increased complication rates (43% vs. 29%), prolonged hospitalization, and the need for more intensive treatment [3]. The location of the duodenum outside the peritoneum (retroperitoneal) often masks clinical signs, making early diagnosis challenging [3]. Nevertheless, appropriate imaging significantly enhances diagnostic accuracy [5].

Over 90% of abdominal traumas in children are managed non-operatively. Surgical intervention is generally reserved for cases involving hemodynamic instability, associated organ injury, imaging evidence of extravasation, or clinical deterioration [6-8]. The American Association for the Surgery of Trauma (AAST) injury scale for the duodenum is a valuable tool for assessing the severity of the injury, demonstrating a strong correlation with the need for surgical intervention [3]. According to this scale, duodenal hematomas are classified as Grade I or Grade II, depending on whether one or multiple portions are involved, respectively. 

Given the diagnostic challenges and the limited literature on pediatric duodenal hematomas, we report the case of a four-year-old girl with an isolated intramural hematoma (between the smooth muscle layer and submucosa) of the duodenum resulting from blunt bicycle handlebar injury.

## Case presentation

Initial presentation

A four-year-old girl presented to the Emergency Department one day after sustaining a fall from a bicycle, during which she struck her right upper abdomen (right hypochondrium) on the handlebar. Her symptoms had worsened over the preceding 24 hours and included nausea, abdominal pain, and brown-colored emesis. An initial ultrasound, performed immediately after the accident, showed no abnormalities.

On admission, the patient was conscious, in verbal and logical contact adequate to her age, and hemodynamically stable. Physical examination revealed the abdomen without resistance but tender. A 1-cm area of ecchymosis was observed in the right mesogastric region. Peristalsis was present and normal.

Diagnostic workup

Laboratory tests revealed a significant elevation in serum lipase and C-reactive protein (CRP) levels (Table 1).

**Table 1:** Values of select parameters recorded throughout the hospitalization period. Hematocrit (HCT) and hemoglobin (HGB) levels decreased during the first few days; however, there were no associated clinical symptoms or indication for transfusion. CRP: C-reactive protein; HCT: hematocrit test; MCH: mean corpuscular hemoglobin; MCHC: mean corpuscular haemoglobin concentration; MCV: mean corpuscular volume; PLT: platelet count.

Parameter		On Admission		On Surgical Unit			Post-treatment day 2			Referential Values for Pediatric Patients		
Biochemical Values												
Lipase (U/l)		64		129			72			<34		
CRP (mg/dl)		1.5		1.49			0.86			<0.5		
Amylase (U/l)		45		86			61			28-100		
Hematological Values												
White blood cell count (10^3^/µL)		8.67		7.3			4.3			4.5-13		
Red blood cell count (10^6^/ µL)		4.83		4.33			3.63			4.3-5.5		
Hemoglobin (HGB) (g/dL)		13.3		11.9			10.2			10.9-14.2		
HCT (%)		38.1		34.1			29.2			34-41		
MCH (pq)		27.5		27.5			28.1			25-31		
MCHC (g/dL)		34.9		34.9			34.9			32-35		
MCV (fL)		78.9		78.8			80.4			76-90		
PLT (10^3^/µL)		238		204			170			150-400		

**Figure 1:**
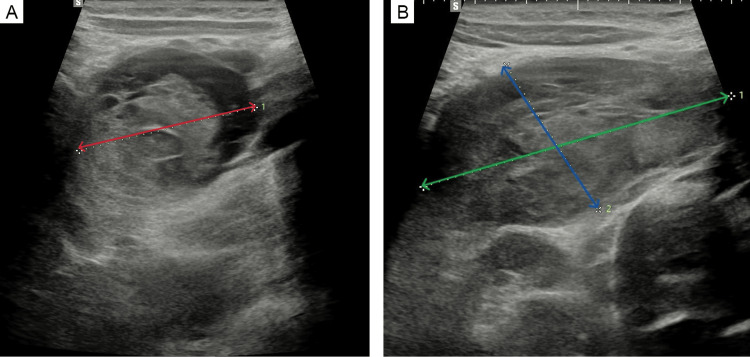
The ultrasound performed on admission Ultrasound image showing large hematoma; measurements: diameter 40.2 mm (A, red arrow), width 33.2 mm (B, blue arrow), length 62.5 mm (B, green arrow). The duodenal lumen was not visualized due to external compression caused by a duodenal hematoma.

Repeat abdominal ultrasound performed upon admission demonstrated a heterogeneous, solid-fluid lesion measuring 62.5x33.2x40.02 mm, located in the retroperitoneal space of mesogastrium consisting of hematoma (Figure 1). The lesion appeared to compress the adjacent segment of the duodenum superiorly. Additionally, the inferior vena cava (IVC) was noted to be flattened.

**Figure 2:**
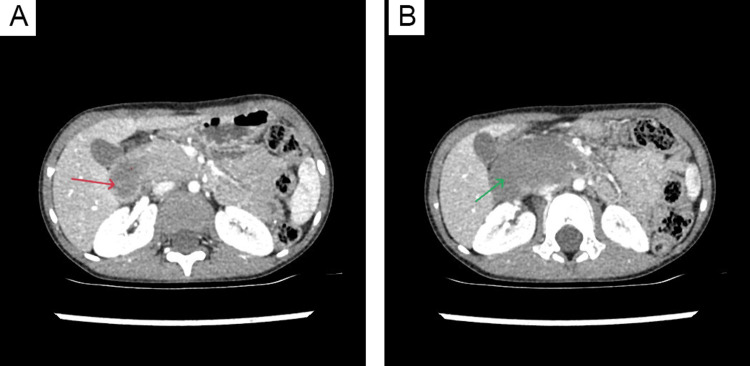
Computed tomography image showing a hematoma compressing the inferior vena cava (A) and the common hepatic duct (B) (A) CT image showing a hematoma at the level of the pancreas (red arrow). (B) CT image showing a hematoma located below the head of the pancreas (green arrow).

A computed tomography (CT) scan confirmed the presence of a heterogeneous lesion located in the region of the pancreatic head, measuring 79x38x39 mm. The lesion caused compression of both the IVC and the common hepatic duct (Figure 2). The CT also revealed narrowing of the duodenum distal to its superior portion. The head of the pancreas was noted to contour the upper-medial surface of the hematoma, raising suspicion that the hematoma may have originated from the pancreas itself or representing intramural duodenal hematoma. 

Treatment course

The patient was admitted to the pediatric surgical unit, where she received intravenous fluids, analgesics, cefuroxime (Bifuroxym), and metronidazole. Following the initiation of treatment, her clinical condition improved, and she no longer reported any abdominal pain. A nasogastric tube was inserted, yielding approximately 60 ml of tea-colored fluid.

Follow-up laboratory tests again demonstrated elevated levels of lipase and CRP (Table 1).

**Figure 3:**
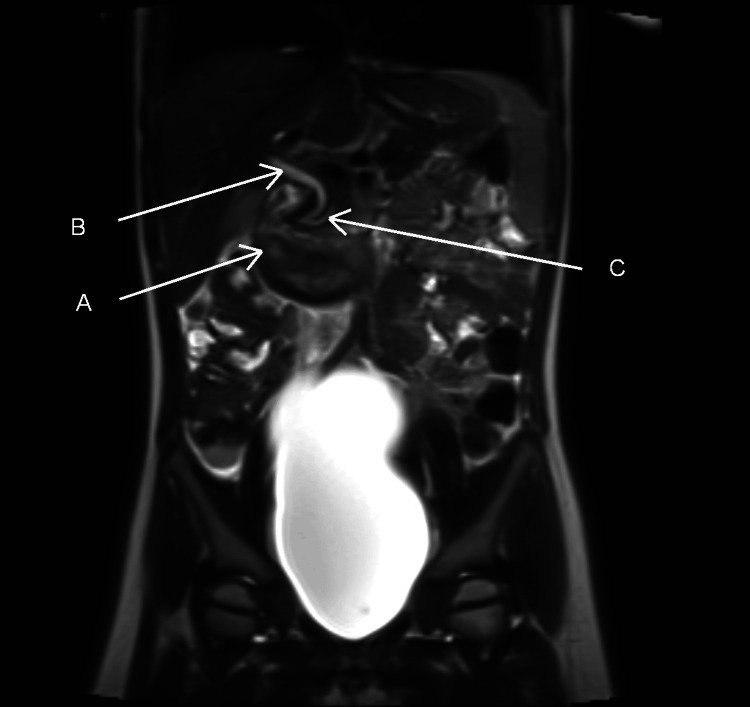
MRI image demonstrating marked dilation of the common bile duct and pancreatic duct due to compression by hematoma (A) White arrow indicating duodenal hematoma; (B) White arrow indicating common bile duct; (C) White arrow indicating pancreatic duct. The anatomical location of both ducts predispose them to dilation caused by external compression from the duodenal hematoma demonstrated in the presented image. MRI reveals contusion of the pancreatic head without evdence of pancreatic duct injury.

To further evaluate for possible duodenal perforation and/or pancreatic duct injury, magnetic resonance imaging (MRI) was performed. The scan excluded duodenal perforation and confirmed that the lesion was an intramural hematoma. The pancreatic duct (measuring 2.5-3 mm) and the common bile duct (3.5-4 mm) were visualized and appeared compressed by the hematoma (Figure 3).

Regarding the pancreas injury, parenteral nutrition, along with Bifuroxym and metronidazole, were included in the treatment process. Two days after the initiation of antibiotics and one day following the start of parenteral nutrition, laboratory tests showed a decline in CRP and pancreatic enzyme levels (Table 1).

**Figure 4:**
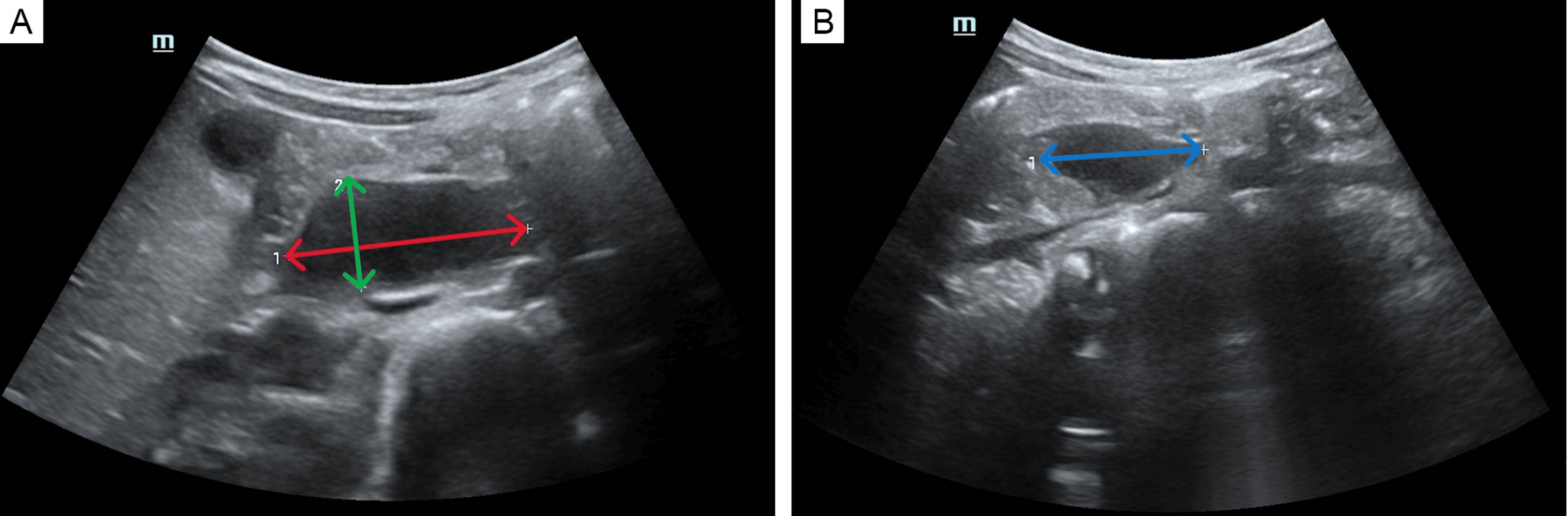
Follow-up ultrasound on ninth day post-admission (A) Ultrasound image showing width of the hematoma (17.6 mm) (green arrow) and length of the hematoma (40.4 mm) (red arrow). (B) Ultrasound image showing the diameter of the hematoma (31.6 mm) (blue arrow).

Treatment was continued for an additional six days, during which nasogastric drainage progressively cleared. Antimicrobial prophylaxis was discontinued on the third day. A follow-up abdominal ultrasound was performed after the treatment period, revealing a reduction in the size of the hematoma, now measuring 40.4x32x17.6 mm (Figure 4).

On the day following the control ultrasound, parenteral nutrition was discontinued and replaced with oral feeding, which the patient tolerated well. The nasogastric tube was removed.

Six days after the initiation of oral nutrition, a repeat abdominal ultrasound demonstrated a continued reduction in hematoma size, measuring 29x9 mm. Given the favorable clinical and radiologic progress, the patient was discharged home.

Follow-up

**Figure 5:**
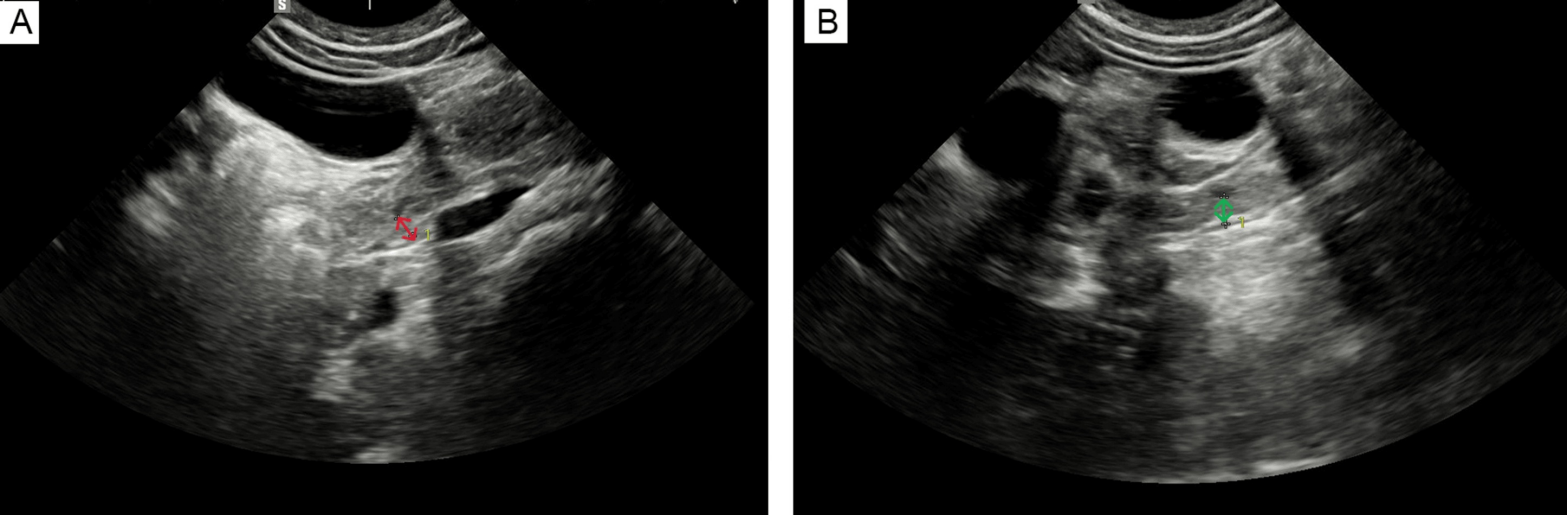
Control ultrasound after four months (A) Red arrow shows measurement of the hematoma (3.4 mm). (B) Green arrow shows measurement of the hematoma (3.1 mm).

At a four-month follow-up visit, ultrasound imaging showed near-complete resolution of the hematoma, with only a small residual lesion measuring 3.1x3.4 mm (Figure 5).

## Discussion

Duodenal hematoma is a rare condition that accounts for approximately 3%-5% of all abdominal injuries in the pediatric population [1]. In children, the most common causes of duodenal hematoma are blunt abdominal traumas, including child abuse, motor vehicle injuries, bicycle accidents, sports-related injuries, auto-pedestrian incidents, and handlebar injuries [3,4,6].

Due to the retroperitoneal location of the duodenum, physical examination findings are often nonspecific, ranging from mild abdominal tenderness to rigidity [3]. Additional clinical manifestations typically include abdominal pain and vomiting [1,4]. Peterson et al. [4] reported that more than half of the pediatric patients with duodenal hematoma in their study had elevated serum lipase and/or amylase levels.

Non-operative management remains the mainstay of treatment for duodenal hematoma in children [3]. This conservative approach generally includes the following key components: prompt diagnosis and excluding ptransmural perforation, evaluation for concomitant pancreatic injury with serum lipase and amylase testing, assessment of nasogastric tube output, bowel rest combined with parenteral nutrition, and serial monitoring of hematoma size via abdominal ultrasound [1,4,9].

Timely diagnosis of duodenal hematoma is essential, as delays exceeding 24 hours may prolong hospitalization and increase the risk of complications, including pancreatitis, sepsis, partial small bowel obstructions, and more [3,4]. Given the nonspecific findings on physical examination, imaging plays a pivotal role in facilitating early diagnosis, which is critical in cases of duodenal injury [3,5].

While ultrasound is widely accessible and non-invasive, its low sensitivity limits its diagnostic utility. Therefore, many authors suggest its use primarily as a screening modality [3,5,10-12]. Notably, Nural et al. [12] advocate for continued clinical observation even in patients with negative ultrasound findings. In line with this approach, Stengel et al. [5] recommend further imaging studies to confirm or exclude injury, which is consistent with our clinical experiance - initial ultrasound in our case failed to detect any abnormalities. 

Computed tomography (CT) is frequently referred to as the ''gold standard” in evaluating blunt abdominal trauma [1,3,4,13-15]. CT is particularly valuable in distinguishing between duodenal hematoma and perforation, as the former often warrants non-operative management, while the latter may require surgical intervention [1,13,14].

Rhea et al. [11] provide a thorough comparison between CT and ultrasound, highlighting the strengths and limitations of each test. He describes CT as the ''test of choice'' for evaluating hemodynamically stable patients with blunt abdominal trauma and acknowledges ultrasound as a useful initial or adjunctive tool, particularly prior to exploratory laparotomy in the setting of significant hemoperitoneum.

Children typically have a greater capacity for recovery from duodenal trauma than adults, making non-operative management more appropriate in the majority of cases [3,6]. As previously mentioned, over 90% of pediatric abdominal traumas are managed non-operatively. Although surgical intervention is rare, Peterson et al. [4] and Clendenon et al. [3] each report one case requiring surgical evacuation due to either hematoma size or persistent obstructive symptoms.

Some studies advocate for surgical intervention if obstruction symptoms persist beyond 10-14 days [15]. However, more recent literature suggests a longer observation period, even six weeks, as more accurate [1].

The AAST injury grading scale is a helpful tool in clinical decision-making [3]. Clendenon et al. [3] found a strong correlation between injury grade and the need for surgical intervention. Another important determinant is hemodynamic stability, as noted by Shimizu et al. [6], although the authors acknowledge that the evaluation of stability may be complicated in cases involving multiple organ injuries and varies depending on the child.

It is important to acknowledge the small sample size in most studies, a limitation resulting from the rarity of duodenal hematoma. Despite this, the literature consistently supports a conservative approach, reserving surgery for patients with additional injuries [7,8] or an atypical course during non-operative management, taking into account hemodynamic status and AAST injury grade.

During non-operative treatment, the timing of reintroducing oral feeding is a critical aspect of recovery. In our case, the patient successfully resumed oral intake on day nine, aligning Shen et al. [1] who reported a median of 17 days (range 9-32 days), Clendenon et al. [3] seven days, and Peterson et al. [4] seven days for grade I AAST injury (range 0-23 days). Despite interpatient variability, three key criteria should be met before initiating oral feeding: absence of abdominal pain, clearing and increasing volume of nasogastric tube output, and conformation of duodenal passage on ultrasound [1].

The current body of literature overwhelmingly supports non-operative management as the standard of care for pediatric patients with duodenal hematoma. The case presented herein reinforces the feasibility and effectiveness of conservative treatment, even in instances of large duodenal hematomas, with satisfying clinical outcomes.

## Conclusions

This case report highlights the successful non-operative management of a large intramural duodenal hematoma in a pediatric patient following blunt abdominal trauma. Despite the diagnostic challenges associated with the retroperitoneal location of the duodenum and the potential for false-negative findings on initial ultrasonography, timely imaging with computed tomography and magnetic resonance imaging enabled an accurate diagnosis and informed therapeutic decision-making. The favorable clinical outcome achieved through conservative treatment, including bowel rest, nasogastric decompression, parenteral nutrition, antibiotic therapy, and serial imaging, supports the growing consensus that non-operative management is appropriate and effective in hemodynamically stable children with isolated duodenal hematomas.

This case underscores the importance of a structured, multidisciplinary approach and reinforces the role of close clinical and radiological monitoring in guiding the transition to oral feeding and discharge. Our management in this case and satisfactory outcomes achieved support the recommendations found in the literature regarding the timing of oral feeding, the role of ultrasound as both a screening and follow-up modality, CT as the gold standard for diagnosing duodenal hematoma, and CT or MRI as a valuable tool for differentiating duodenal hematoma from duodenal perforation.

Given the rarity of duodenal hematoma in children and the limited number of cases reported in the literature, an individualized approach to management is warranted. This case may provide useful reference to guide clinical decision-making in similar scenarios.
